# Assessing the ability of ChatGPT 4.0 in generating check-up reports

**DOI:** 10.3389/fmed.2025.1658561

**Published:** 2025-10-07

**Authors:** Yikai Chen, Yuxin Liu, Yuanchang Huang, Xiujie Huang, Zhuoqun Zheng, Fangjie Yang, Haiming Lin, Haoyu Lin, Xinxin Li, Aosi Xie, Yiteng Huang

**Affiliations:** ^1^Department of Gastroenterological Surgery, The First Affiliated Hospital of Shantou University Medical College, Shantou, China; ^2^Health Care Center, The First Affiliated Hospital of Shantou University Medical College, Shantou, China; ^3^Department of Orthopaedics, The First Affiliated Hospital of Shantou University Medical College, Shantou, China; ^4^Faculty of Medicine and Dentistry, School of Dentistry, University of Alberta, Edmonton, AB, Canada; ^5^Department of Thyroid and Breast Surgery, The First Affiliated Hospital of Shantou University Medical College, Shantou, China

**Keywords:** health care service, artificial intelligence, check-up, ChatGPT 4.0, health report

## Abstract

**Background:**

ChatGPT (Chat Generative Pre-trained Transformer), a generative language model, has been applied across various clinical domains. Health check-ups, a widely adopted method for comprehensively assessing personal health, are now chosen by an increasing number of individuals. This study aimed to evaluate ChatGPT 4.0’s ability to efficiently provide patients with accurate and personalized health reports.

**Methods:**

A total of 89 check-up reports generated by ChatGPT 4.0 were assessed. The reports were derived from the Check-up Center of the First Affiliated Hospital of Shantou University Medical College. Each report was translated into English by ChatGPT 4.0 and graded independently by three qualified doctors in both English and Chinese. The grading criteria encompassed six aspects: adherence to current treatment guidelines (Guide), diagnostic accuracy (Diagnosis), logical flow of information (Order), systematic presentation (System), internal consistency (Consistency), and appropriateness of recommendations (Suggestion), each scored on a 4-point scale. The complexity of the cases was categorized into three levels (LOW, MEDIUM, HIGH). Wilcoxon rank sum test and Kruskal-Wallis test were selected to examine differences in grading across languages and complexity levels.

**Results:**

ChatGPT 4.0 demonstrated strong performance in adhering to clinical guidelines, providing accurate diagnoses, systematic presentation, and maintaining consistency. However, it struggled with prioritizing high-risk items and providing comprehensive suggestions. In the “Order” category, a significant proportion of reports contained mixed data, several reports being completely incorrect. In the “Suggestion” category, most reports were deemed correct but inadequate. No significant language advantage was observed, with performance varying across complexity levels. English reports showed significant differences in grading across complexity levels, while Chinese reports exhibited distinct performance across all categories.

**Conclusion:**

In conclusion, ChatGPT 4.0 is currently well-suited as an assistant to the chief examiner, particularly for handling simpler tasks and contributing to specific sections of check-up reports. It holds the potential to enhance medical efficiency, improve the quality of clinical check-up work, and deliver patient-centered services.

## Background

In the rapidly evolving landscape of healthcare, the integration of AI represents a significant advancement. ChatGPT (Chat Generative Pre-trained Transformer), developed by OpenAI and released on November 30, 2022, is a cutting-edge generative language model trained with Reinforcement Learning from Human Feedback (RLHF). Its deep learning architecture allows ChatGPT to assimilate vast amounts of data, aligning with the capabilities of artificial general intelligence, enabling it to intelligently acquire and process up-to-date information, and interact with users conversationally (1–3). ChatGPT can understand user inputs distinctly and accurately using its AI-based deep learning model (3). As an advanced assistant designed to aid humanity, there is growing interest in its potential applications in the medical field (1).

Extensive research has demonstrated the considerable potential of artificial intelligence (AI) in accelerating scientific development and improving scientific literacy, particularly in medical research. ChatGPT has shown remarkable capabilities in various tasks such as experiments, scientific writing, and information retrieval (4–6). Consequently, ChatGPT is highly anticipated to contribute to clinical diagnosis and treatment (2). Numerous studies have shown that ChatGPT has been involved in various clinical trials, including General Surgery, Dentistry, and Plastic and Reconstructive Surgery, proving its potential in assisting both scientific researchers and healthcare professionals (7–10).

Research has confirmed ChatGPT’s remarkable ability to handle complex data efficiently, potentially reducing the time needed to manage various tasks (4–6). While it does not interact directly with patients, ChatGPT can complete various essential tasks such as summarizing medical histories, evaluating investigations, and categorizing clinical parameters, playing a vital role in assisting diagnosis and guiding treatment (4, 5) Despite facing certain limitations, such as legislative restrictions and ethical issues in clinical practice, there is still substantial potential for AI applications to be explored. ChatGPT 4.0, the latest version, offers astonishing precision and steerability, promising greater performance in specialized fields (1). Its application potential in clinical settings has been affirmed and its ability to evaluate and write medical reports is being discovered (3, 7, 11).

Health check-ups are one of the most effective methods for comprehensively understanding an individual’s health status, significantly impacting healthcare provision (12). With the growing awareness of overall health and advancements in clinical technology, the public is increasingly attentive to their physical well-being and more inclined to undergo health check-ups to evaluate their health, identify diseases, and seek early treatment (13, 14). Traditionally, healthcare professionals play critical roles in diagnosing, prescribing medications, providing health advice, and writing medical reports (14). The reports generated by check-up doctors, along with their therapeutic decisions and lifestyle recommendations, are considered authoritative. However, there is a growing demand for more efficient and accurate reports due to the increasing workload faced by chief examiners (10, 15). In this situation, we consider attempting to combine the potential ability of ChatGPT and generation of check-up reports. We explore whether ChatGPT is qualified as the chief check-up doctor, alleviating the increasing workload of them and providing patients with more accurate and personalized check-up reports more efficiently.

The study was approved by the Ethics Committee of the First Affiliated Hospital of Shantou University Medical College. Informed consent of participants was approved to be waived.

## Methods

### Data

**Figure 1:**
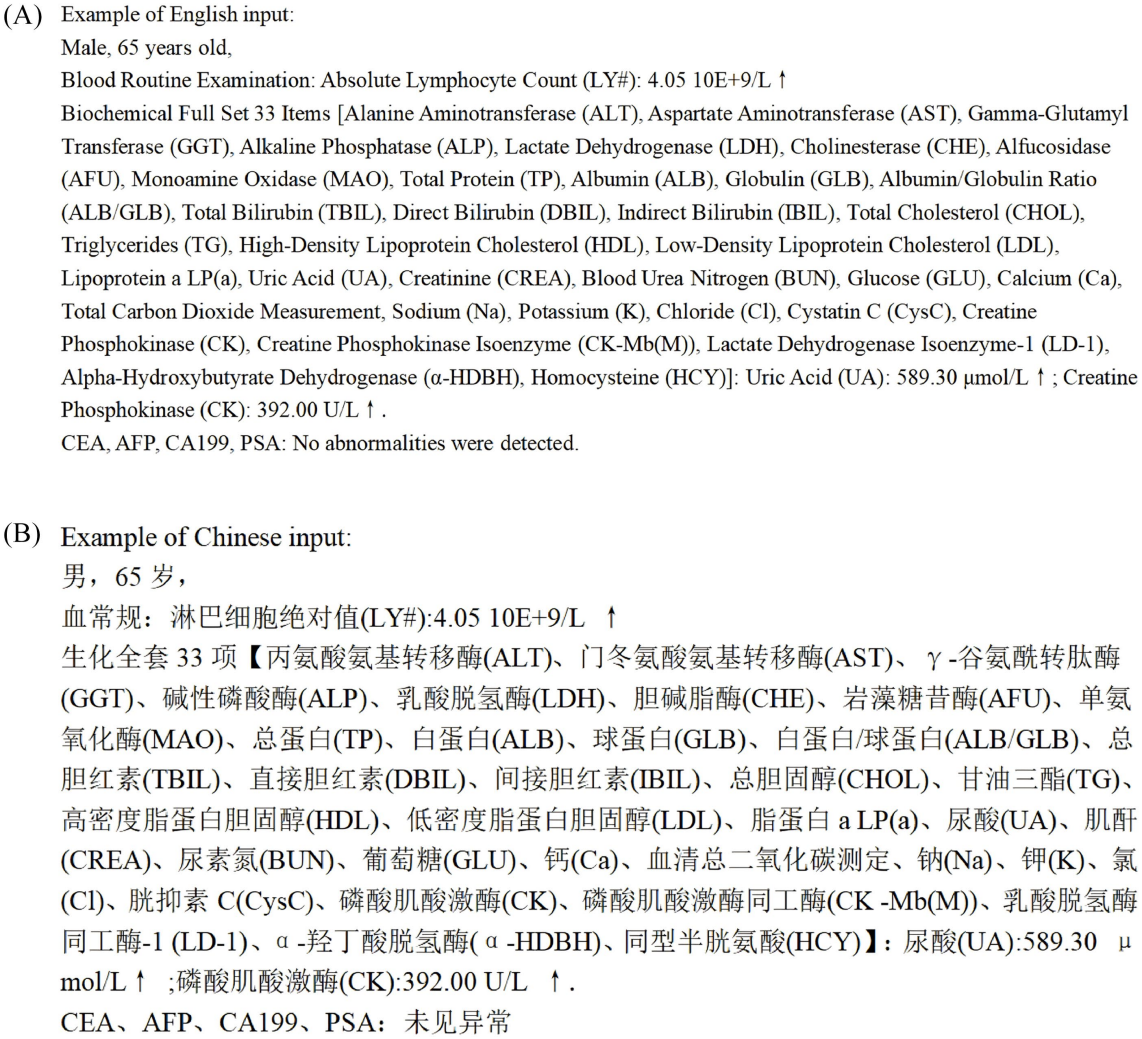
**(A)** Example of English input format. **(B)** Example of Chinese input format.

We analyzed 89 check-up cases collected from the Check-up Center of the First Affiliated Hospital of Shantou University Medical College, randomly selected from the database for 2021–2023. Data extracted from these 89 reports included gender, age, and check-up items (Figure 1). To ensure patient confidentiality and adhere to privacy regulations, all personally identifiable information, such as patients’ names, was removed, and de-identified data were used throughout the study (1).

All patient data were translated into English at the same translating level to test the samples in both Chinese and English, allowing us to observe ChatGPT 4.0’s performance in different languages. The translation tasks were executed by ChatGPT 4.0, with all translations being carried out within the same dialog box to ensure consistency in translation quality. The 89 samples were divided into three groups (LOW, MEDIUM, and HIGH) based on their complexity. The number of abnormal results was used as the criterion to objectively reflect complexity. Samples with fewer than 4 abnormal results were classified as LOW, those with 4–8 abnormal results as MEDIUM, and those with 9 or more abnormal results as HIGH. Twenty-seven samples were categorized in group LOW, and group MEDIUM and HIGH had 31 samples each. Subsequently, we compared the performance of ChatGPT 4.0 within these groups.

### Prompt engineering

**Figure 2:**
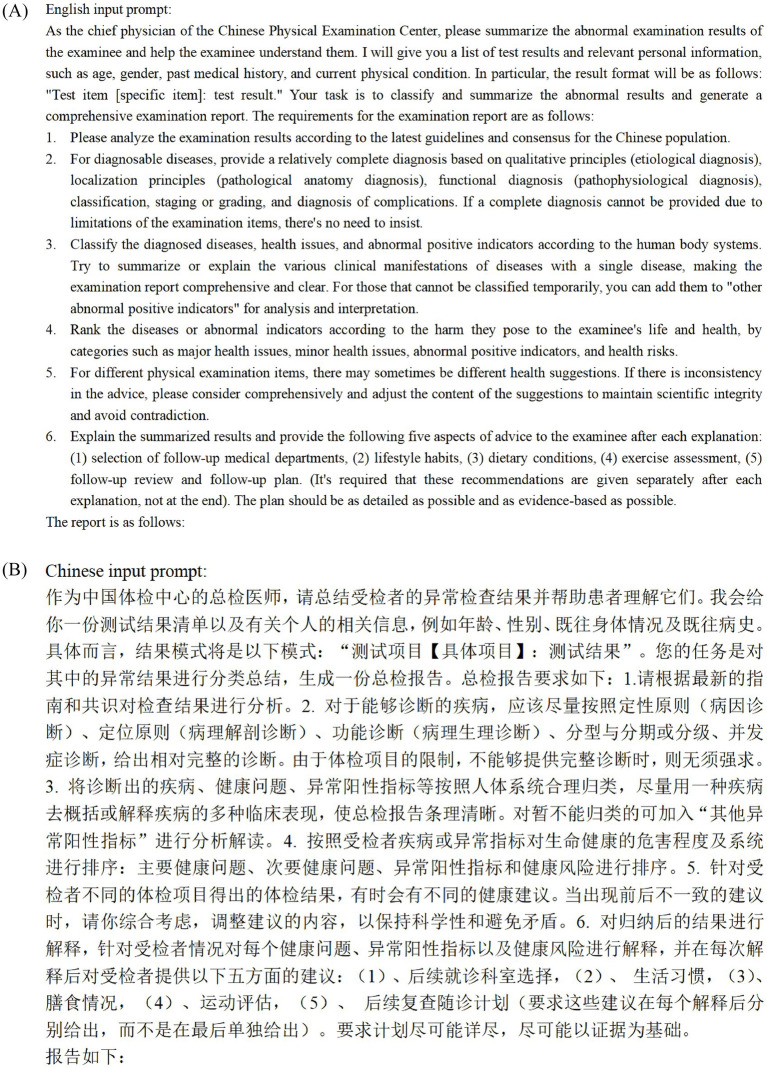
**(A)** English input prompt. **(B)** Chinese input prompt.

The input format of the dialog was standardized according to the descriptions of each examination or investigation item provided by the Check-up Center. We avoided the use of multiple names for the same item in the reports input to ChatGPT, such as “blood glucose” and “blood sugar.” Additionally, we standardized the units for each laboratory test indicator in our study, for example, using only “mmHg” as the unit for blood pressure measurements. The inconsistency rate of terminology in the data was found to be less than 1%, as verified by two independent researchers. These measures were taken to prevent errors arising from inconsistencies in the data (16). After multiple iterations of testing, the final input directive (Figure 2) was refined for clarity and accuracy, ensuring consistent data formatting for ChatGPT 4.0.

No additional pre-training was conducted in the study. Notably, all samples were examined separately in individual dialog boxes, and only the first answers were considered to prevent ChatGPT 4.0 from learning and improving its responses through repeated interactions.

### Grading

**Figure 3:**
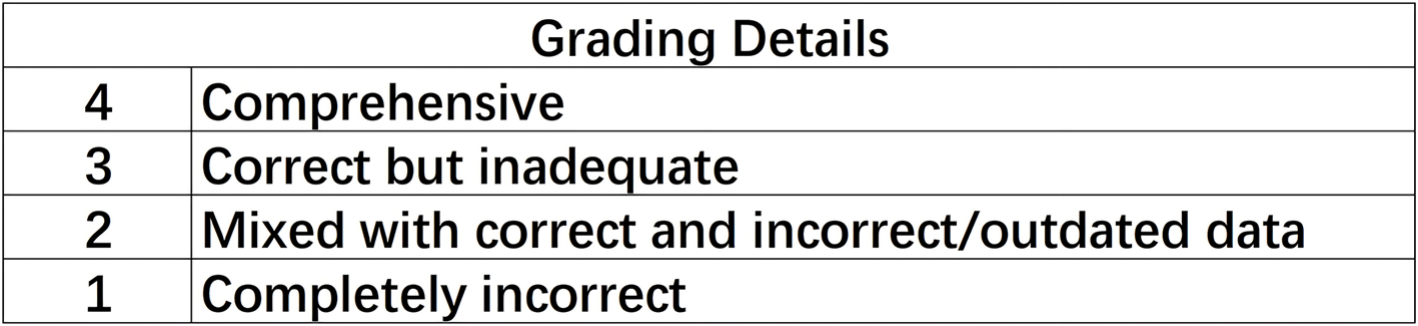
Grading details.

Three qualified doctors from the first Affiliated Hospital of Shantou University Medical College participated in assessing the check-up reports generated by ChatGPT 4.0. All the doctors were blinded to whether they were reviewing ChatGPT- or human-generated check-up reports. Evaluators used a standardized grading rubric to assess six criteria, each scored on a 4-point scale: adherence to current treatment guidelines (Guide), diagnostic accuracy (Diagnosis), logical flow of information (Order), systematic presentation (System), internal consistency (Consistency), and appropriateness of recommendations (Suggestion). All the evaluation criteria are strictly formulated in accordance with the latest authoritative guidelines in the field of health checkups in China, namely the Expert Consensus on the Chief Physician Report for Health Checkupand the Expert Consensus on Basic Items of Health Checkup (17, 18). The scoring system was as follows: 1 = Completely incorrect, 2 = Mixed with correct and incorrect/outdated data, 3 = Correct but inadequate, 4 = Comprehensive (Figure 3) (19).

To ensure inter-rater reliability, a preliminary calibration round was conducted, followed by a consensus meeting to align the evaluators’ understanding of the grading rubric. Each of the six items in each report was graded. In cases of assessment discrepancies, the senior chief doctor, with over 10 years of experience in the medical check-up center, made the final decision and provided the ultimate grade. Evaluators also summarized the advantages and disadvantages of the responses provided by ChatGPT 4.0 and proposed specific points for improvement (17–21).

### Statistical analysis

All responses generated by ChatGPT 4.0 were recorded using Microsoft Office Word 2016, and the grades given by evaluators were documented in Microsoft Office Excel 2016. Data analyses were executed using IBM SPSS Statistics 21. The percentage distribution of different grades for each item was calculated to illustrate the detailed grading situation of ChatGPT 4.0’s responses across the six criteria.

Wilcoxon rank sum test was used to examine differences in grade situations between different language groups, with a *p* value < 0.05 considered significant. The Kruskal-Wallis test was selected to examine differences in grading among the three complexity levels. Mean ranks were used to compare grading situations among the three groups, after Bonferroni correction, with a *p* value < 0.0167 considered significant. For *post-hoc* tests following a rank sum test, the Bonferroni correction was applied to adjust the *p*-value. Ultimately, the utilization of confidence intervals and effect sizes serves to substantiate the significance of the observed differences and thereby augment the robustness of the statistical findings. All statistical and analytical tasks were completed by a junior doctor, independent of the three evaluators.

## Results

### Evaluation of check-up reports generated by ChatGPT 4.0

**Figure 4:**
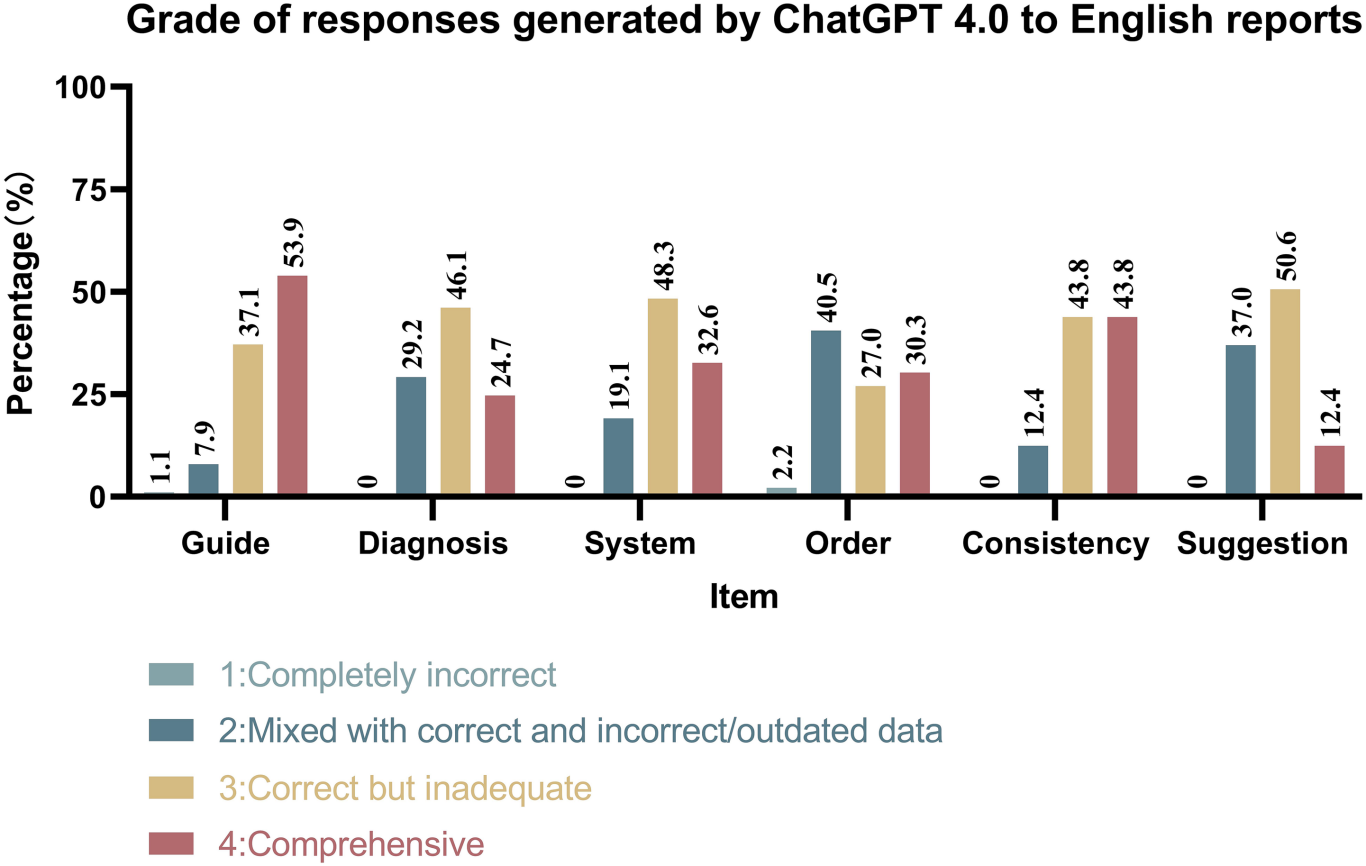
Grade of responses generated by ChatGPT 4.0 to English reports.

**Figure 5:**
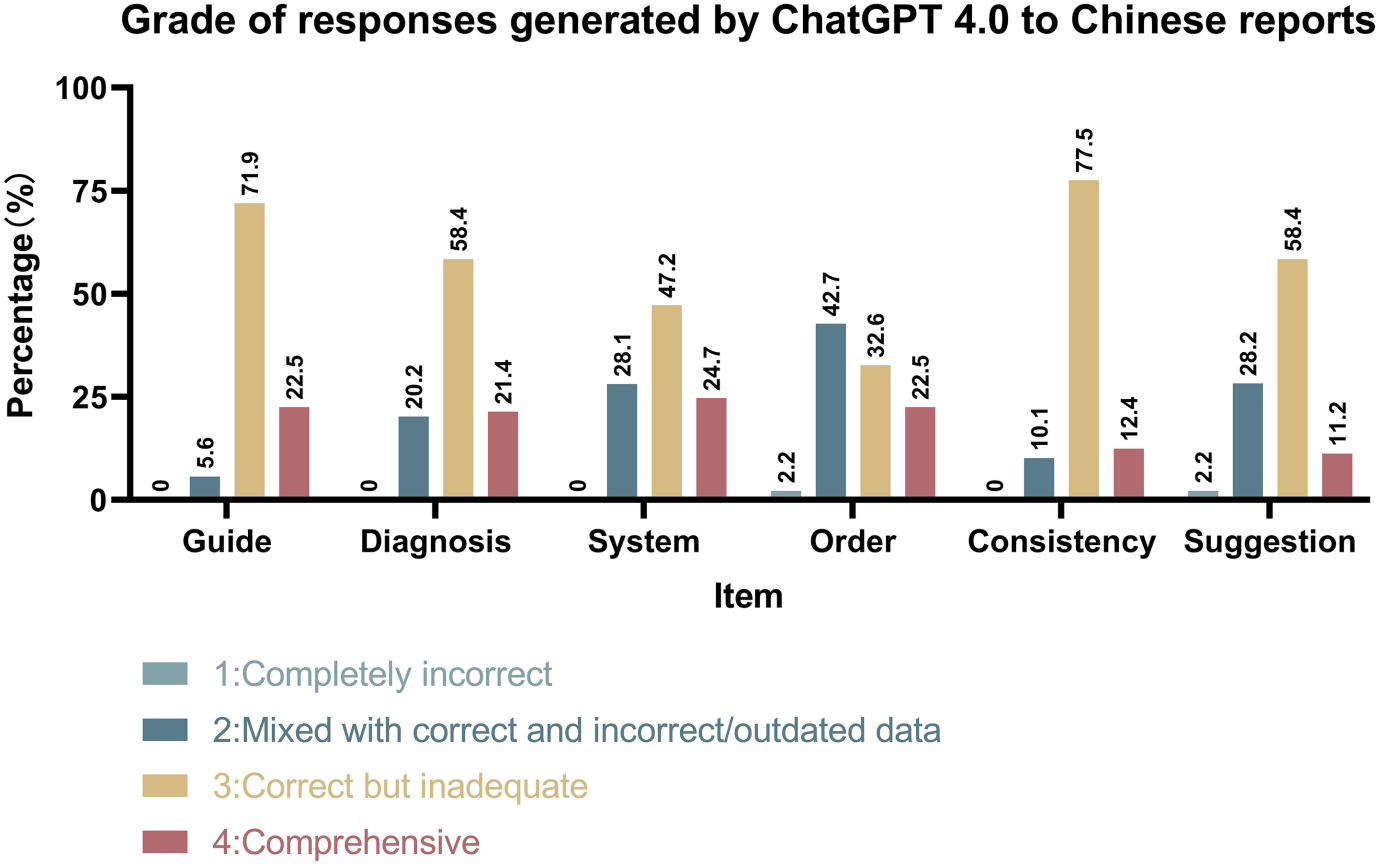
Grade of responses generated by ChatGPT 4.0 to Chinese reports.

As illustrated in Figures 4, 5, ChatGPT 4.0 demonstrated excellent performance in generating check-up reports in both English and Chinese cases. ChatGPT 4.0 exhibited outstanding competence in adhering to clinical guidelines, providing accurate diagnoses, systematic presentation, and internal consistency. In all four categories, the proportion of reports deemed correct but inadequate or comprehensive was the highest, with the combined percentage of these two types of reports exceeding 70% in each category. Notably, only one report was completely incorrect in the “Guide” category for Chinese reports, and no other incorrect reports were found in either language for the four categories mentioned.

However, ChatGPT 4.0 did not perform as well in prioritizing check-up items based on risk factors and providing satisfactory medical suggestions. In the “Order” category, reports mixed with correct and incorrect/outdated data had the largest proportion. Additionally, 2.2% of English reports and 2.2% of Chinese reports were considered completely incorrect, while 30.3% of English reports and 22.5% of Chinese reports received the highest rating. In the “Suggestion” category, most reports were assessed as correct but inadequate or mixed with correct and incorrect/outdated data, with 2.25% of Chinese reports being completely incorrect.

### Comparison of different languages and complexity levels

As depicted in Tables 1–3, when confronted with cases of varying complexity levels, English and Chinese reports were observed to outperform each other in certain categories. In LOW complexity cases, no significant difference was observed between English and Chinese reports in the “Order,” “Consistency,” and “Suggestion” categories, with Chinese reports receiving higher grades in the remaining items. In MEDIUM complexity cases, English and Chinese reports received similar grades in the “Diagnosis,” “System,” and “Consistency” categories, with English reports graded better in “Consistency.” In HIGH complexity cases, Chinese reports received similar grades in most items as English reports but performed better in “System,” and “Suggestion.”

**Table 1:** Grade of responses generated by ChatGPT 4.0 to reports in different languages in LOW complexity cases.

Complexity	Items	Wilcoxon *W* value	*p*-value	Rank-Biserial *r*	95%CI for *r*
LOW	**Guide**	464.5	0.003	**0.47**	[0.17, 0.77]
	**Diagnosis**	523.0	<0.001	**0.59**	[0.33, 0.85]
	**System**	492.0	<0.001	**0.54**	[0.26, 0.82]
	Order	424.0	0.23	0.19	[−0.12, 0.50]
	Consistency	392.5	0.71	0.06	[−0.25, 0.37]
	Suggestion	441.5	0.09	0.27	[−0.04, 0.58]

Bold values indicate those that exhibit significant differences in the comparison.

**Table 2:** Grade of responses generated by ChatGPT 4.0 to reports in different languages in MEDIUM complexity cases.

Complexity	Items	Wilcoxon *W* value	*p*-value	Rank-Biserial *r*	95%CI for *r*
MEDIUM	**Guide**	628.5	0.001	**−0.40**	[−0.63, −0.17]
	Diagnosis	550.5	0.94	−0.01	[−0.26, 0.24]
	**System**	527.0	0.53	−0.09	[−0.34, 0.16]
	**Order**	577.0	0.02	**−0.31**	[−0.56, −0.06]
	Consistency	501.0	0.27	−0.16	[−0.41, 0.09]
	**Suggestion**	458.5	0.003	**−0.41**	[−0.64, −0.18]

Bold values indicate those that exhibit significant differences in the comparison.

**Table 3:** Grade of responses generated by ChatGPT 4.0 to reports in different languages in HIGH complexity cases.

Complexity	Items	Wilcoxon *W* value	*p*-value	Rank-Biserial *r*	95%CI for *r*
HIGH	Guide	524.5	0.92	0.01	[−0.24, 0.26]
	Diagnosis	554.5	0.21	0.17	[−0.08, 0.42]
	**System**	462.0	0.02	**0.31**	[0.06, 0.56]
	Order	536.5	0.57	0.07	[−0.18, 0.32]
	Consistency	495.5	0.38	0.12	[−0.13, 0.37]
	**Suggestion**	435.5	0.009	**0.35**	[0.10, 0.60]

Bold values indicate those that exhibit significant differences in the comparison.

Tables 4, 5 indicate that significant differences were observed in most categories between grades of English reports across different complexity levels, while Chinese reports showed distinguishing grades across all items for different complexity levels. When generating English reports, ChatGPT 4.0 performed better in LOW complexity cases in almost all the items than in HIGH complexity cases, except “Suggestion,” in which item there were no remarkable differences. For Chinese reports, those generated for LOW complexity cases were considered more comprehensive across all categories. However, there is no significant difference between most cases classified as MEDIUM and HIGH complexity cases, for both English and Chinese reports.

**Table 4:** Grade of responses generated by ChatGPT 4.0 to English reports in different complexity cases.

Language	Items	Comparison	*p*-value	Rank-Biserial *r*	95%CI for *r*
English	Guide	**HIGH vs. LOW**	0.0012	−0.50	[−0.71, −0.29]
		HIGH vs. MEDIUM	0.21	−0.19	[−0.42, 0.04]
		**MEDIUM vs. LOW**	0.022	−0.36	[−0.59, −0.13]
	Diagnosis	**HIGH vs. LOW**	0.0015	−0.49	[−0.70, −0.28]
		HIGH vs. MEDIUM	1.0	−0.02	[−0.25, 0.21]
		**MEDIUM vs. LOW**	0.0023	−0.46	[−0.67, −0.25]
	System	HIGH vs. LOW	0.057	−0.33	[−0.54, −0.12]
		HIGH vs. MEDIUM	1.0	−0.06	[−0.29, 0.17]
		**MEDIUM vs. LOW**	0.022	−0.36	[−0.59, −0.13]
	Order	**HIGH vs. LOW**	<0.0001	−0.64	[−0.79, −0.49]
		HIGH vs. MEDIUM	0.12	−0.23	[−0.46, 0.00]
		**MEDIUM vs. LOW**	0.0002	−0.55	[−0.72, −0.38]
	Consistency	**HIGH vs. LOW**	0.0002	−0.55	[−0.72, −0.38]
		HIGH vs. MEDIUM	0.75	−0.06	[−0.29, 0.17]
		**MEDIUM vs. LOW**	0.0007	−0.51	[−0.70, −0.32]
	Suggestion	HIGH vs. LOW	0.051	−0.34	[−0.55, −0.13]
		HIGH vs. MEDIUM	1.0	−0.08	[−0.31, 0.15]
		MEDIUM vs. LOW	0.11	−0.27	[−0.50, −0.04]

Bold values indicate those that exhibit significant differences in the comparison.

**Table 5:** Grade of responses generated by ChatGPT 4.0 to Chinese reports in different complexity cases.

Language	Items	Comparison	*p*-value	Rank-Biserial *r*	95%CI for *r*
Chinese	Guide	**HIGH vs. LOW**	<0.0001	−0.62	[−0.77, −0.47]
		HIGH vs. MEDIUM	1.0	0.03	[−0.20, 0.26]
		**MEDIUM vs. LOW**	<0.0001	−0.60	[−0.75, −0.45]
	Diagnosis	**HIGH vs. LOW**	<0.0001	−0.70	[−0.82, −0.58]
		**HIGH vs. MEDIUM**	0.019	−0.30	[−0.49, −0.11]
		**MEDIUM vs. LOW**	<0.0001	−0.55	[−0.70, −0.40]
	System	**HIGH vs. LOW**	<0.0001	−0.74	[−0.85, −0.63]
		**HIGH vs. MEDIUM**	0.0002	−0.46	[−0.63, −0.29]
		**MEDIUM vs. LOW**	<0.0001	−0.51	[−0.66, −0.36]
	Order	**HIGH vs. LOW**	<0.0001	−0.67	[−0.80, −0.54]
		HIGH vs. MEDIUM	0.11	−0.20	[−0.39, −0.01]
		**MEDIUM vs. LOW**	<0.0001	−0.61	[−0.76, −0.46]
	Consistency	**HIGH vs. LOW**	0.0005	−0.47	[−0.64, −0.30]
		HIGH vs. MEDIUM	1.0	−0.05	[−0.28, 0.18]
		**MEDIUM vs. LOW**	0.009	−0.38	[−0.57, −0.19]
	Suggestion	**HIGH vs. LOW**	<0.0001	−0.58	[−0.73, −0.43]
		HIGH vs. MEDIUM	0.86	−0.04	[−0.27, 0.19]
		**MEDIUM vs. LOW**	<0.0001	−0.57	[−0.72, −0.42]

Bold values indicate those that exhibit significant differences in the comparison.

## Discussion

The potential of ChatGPT in clinical practice has been a topic of considerable interest, and its capabilities have been tested across various specialties (2, 6–9, 15). ChatGPT has proven to be useful in responding to medical-related questions and aiding doctors and patients in decision-making (14, 22). However, its application in the domain related to check-ups remains limited. This study is a pioneering attempt to apply ChatGPT 4.0 in the check-up area, evaluating its competence in acting as a chief check-up doctor, its capacity to compile check-up reports, and identifying its practical limitations.

We assessed ChatGPT 4.0’s ability to generate check-up reports across multiple dimensions: adherence to the latest clinical guidelines, accuracy of diagnoses, systematic analysis, prioritization of high-risk health items, consistency, and provision of appropriate suggestions. Additionally, we compared the quality of English and Chinese reports and examined performance across different complexity levels. Through rigorous experimental design, careful execution, and meticulous data analysis, we found that ChatGPT 4.0 exhibited outstanding performance in analyzing clinical cases and generating check-up reports (19).

Health check-ups are crucial for identifying health risks, facilitating preventive treatment, and providing lifestyle advice, making the quality of check-up reports vital (14, 23, 24). According to the Expert Consensus on the Chief Physician Report for Health Checkup, the authority principle mandates strict adherence to the latest clinical guidelines, expert consensus, or textbooks in check-up reports. In our study, most reports were evaluated as comprehensive or correct but inadequate, with only one Chinese report receiving the lowest grade of level 1 in the “Guide” category. Additionally, ChatGPT 4.0 performed well in the “Diagnosis,” “System,” and “Consistency” categories. These results demonstrate ChatGPT 4.0’s strong ability to refer to the latest clinical guidelines, provide reliable diagnoses, summarize materials systematically, and maintain a high degree of consistency. Based on these findings and previous experiments on ChatGPT’s clerical capabilities, we are confident that ChatGPT 4.0 can assist doctors in making diagnoses and generating systematic, consistent check-up reports. All clerical work would be performed rigorously following the latest medical guidance. As the pressure on medical professionals increases, check-up doctors not only face a growing number of examination clients but also bear a heavy load of analytical work and paperwork. With the assistance of ChatGPT 4.0, more qualified and accurate reports can be produced in a shorter time. This innovation is expected to significantly improve the efficiency of check-up departments and strengthen the doctor-patient relationship (5, 14, 20, 25).

However, the reports generated by ChatGPT 4.0 show deficiencies in the “Order” and “Suggestion” categories, despite partial reports can still maintain high quality. In both Chinese and English reports, some were considered completely incorrect in terms of order. Evaluators’ feedback revealed that ChatGPT 4.0 exhibits difficulty in consistently maintaining a high degree of orderliness and effectively prioritizing high-risk items within check-up reports. This implies that readers without a medical background are not only unable to readily identify the most noteworthy items in check-up reports at first glance, but also need to devote additional time to carefully review and comprehend each result and recommendation within the reports. It also lacks the skill to provide satisfactory medical recommendations based on a comprehensive patient condition. We believe that these issues may stem from ChatGPT 4.0 operating in a fixed-response mode due to rigid instructions. Further experiments are needed to explore methods for improving report organization and patient-centered medical suggestions.

**Figure 6:**
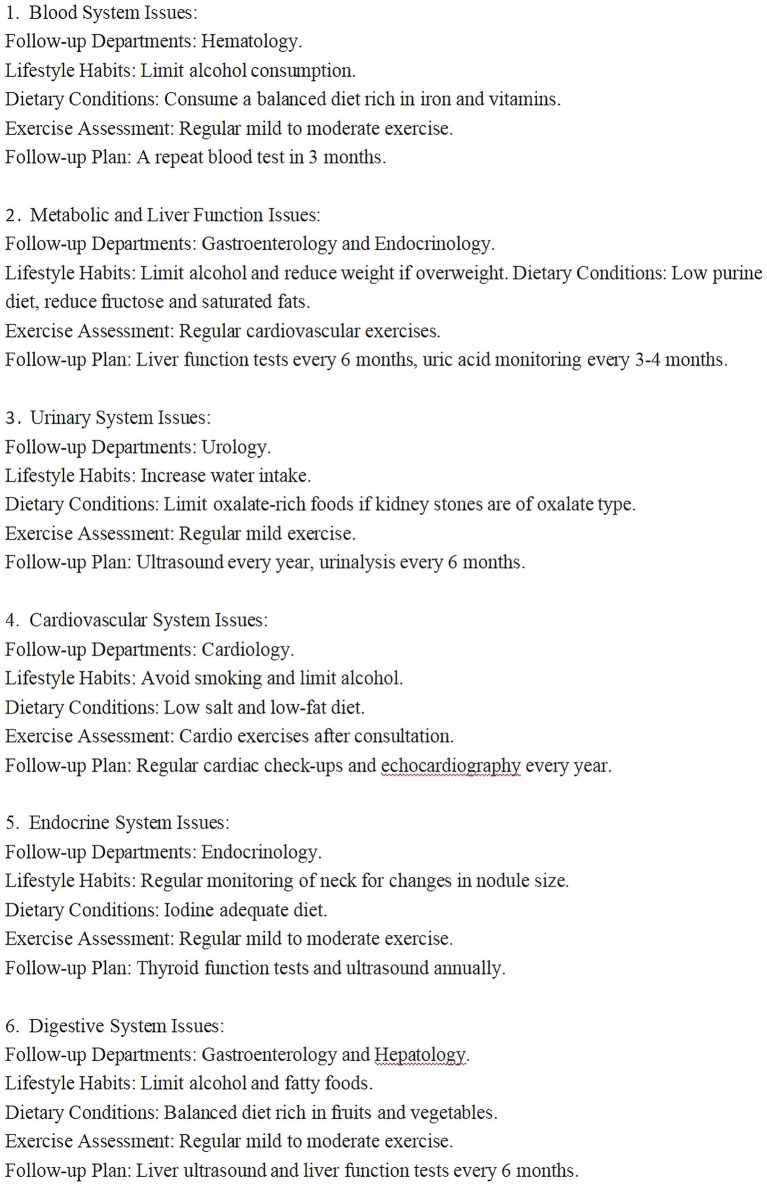
One example of reports with inadequate personalization of suggestions.

Figure 6 illustrates a health examination report with inadequate personalization of recommendations. According to reviewer feedback, the report itemized suggestions for each system and recommended follow-up specialties separately, but failed to consider the interrelationships and common etiologies among these abnormal indicators. For instance, it did not mention the potential metabolic syndrome reflected by multiple abnormal indicators. Moreover, the report did not prioritize which issues were most urgent or posed the highest risk. For example, in the case of a 43-year-old gentleman with a thyroid nodule (TI-RADS category 3) and a hypoechoic liver nodule, both of which require further clarification of their nature, the report did not emphasize their potential malignancy and urgency. Additionally, the report lacked clear explanations, such as specific upper limits for alcohol intake, definitions of low-salt, low-fat, and low-purine diets, and definitions of exercise intensity, which are necessary to guide the examination client in adopting appropriate lifestyle modifications for their age group. This type of report was deemed mixed with correct and incorrect/outdated data.

We anticipate that ChatGPT 4.0 could combine information such as the patient’s age and gender, further integrate multidisciplinary assessments to provide more comprehensive evaluations, rather than discussing different systems in isolation. We believe that qualified personalized recommendations should highlight the most critical health issues for the examination client and provide detailed guidance for further diagnosis and treatment, while refining the definitions of each suggestion, instead of merely proposing vague recommendations such as “control diet” (19).

Additionally, our analysis of ChatGPT 4.0’s performance across different languages and case complexities provided important insights. For medium-complexity cases, the quality of English reports was marginally better than Chinese reports. In contrast, for low- and high-complexity cases, Chinese reports received higher ratings, with variation in specific items. These findings suggest that ChatGPT 4.0’s performance differs depending on the language environment, without a clear overall advantage for either Chinese or English cases. When confronted with English medical cases of varying complexity levels, the performance of ChatGPT 4.0 fluctuated across most aspects, with the exception of the “Suggestion” domain. Specifically, ChatGPT 4.0 demonstrated superior performance when dealing with simple cases. However, its proficiency diminished when encountering cases of moderate or high complexity. In generating Chinese reports, ChatGPT 4.0’s performance was also notably less stable as case complexity changed. This indicates that, ChatGPT 4.0’s ability to comprehensively assess complex medical indicators in both English and Chinese cases fluctuates. According to prior research, OpenAI predominantly trained ChatGPT using English resources, which limited its stability in responding to Chinese queries, particularly those related to health check-ups. Moreover, the differences in background, culture, medical guidelines, and relevant legislation between China and English-speaking countries such as the United Kingdom and the United States pose additional challenges for ChatGPT. Fully understanding the nuances of the Chinese language remains a significant challenge, which affects its performance in complex Chinese medical cases. Concurrently, the medical check-up cases utilized in our study were sourced exclusively from China, characterized by a distinct regional pattern in terms of check-up protocols and documentation methods. This idiosyncrasy may potentially contribute to the diminished performance of ChatGPT 4.0 when confronted with more complex cases (26, 27). Although Chinese reports were rated higher than English reports in more complex cases, it is premature to conclude that ChatGPT 4.0 can fully manage complicated cases. A more cautious conclusion is that ChatGPT 4.0 is better suited to and more experienced in handling simpler cases. And the instability in complex cases makes it difficult to definitively determine which language environment ChatGPT 4.0 excels in.

Based on the above analysis of the experimental results, we affirm that ChatGPT 4.0 possesses a strong capability to process and objectively analyze patient data under fixed instructions or specific conditions. It effectively completes basic tasks, including referring to medical guidelines, providing accurate diagnoses, summarizing issues across different systems, and maintaining the consistency of check-up reports according to the given instructions. We are pleased to observe the proficiency of ChatGPT 4.0 in intelligent summarization and clerical tasks within the medical domain. However, ChatGPT 4.0’s performance is influenced by various objective factors, such as version updates, language types, differences in input instructions, and changes in the database. These factors also impact its ability to provide personalized health guidance to patients. When generating check-up reports, the order of items is often inconsistent, and high-risk results are not always prioritized. This inconsistency is a significant reason why ChatGPT 4.0 is not yet qualified to independently generate quantitative check-up reports. Further testing and refinement of instructions are required to address this issue effectively (4, 5, 10, 15).

While ChatGPT 4.0 shows tremendous potential in clinical work, legal and ethical considerations, such as copyright infringement, medico-legal complications, and privacy concerns, must be addressed (5, 6, 11). These issues currently limit its widespread application in clinical settings.

To implement ChatGPT 4.0 in clinical check-ups, the following key points must be addressed:

1. Improvement of relevant laws and ethics.
2. Careful protection of patient privacy, avoiding the input of private information when issuing instructions.
3. Standardization of various check-up items and units, adhering to a unified format for input content.
4. Enhanced training to ensure high-quality execution of instructions, organized responses, and prioritization of significant health issues (20).
5. Strengthen ChatGPT’s learning of different languages, especially Chinese medical background, medical policy and characteristics (26, 27).

There are a few limitations to this study. First, the sample size may exert an influence on the study outcomes. This study employed data from a single center, which potentially entails limitations such as a small sample size and a homogeneous population of examination clients. In future research, we may enhance the persuasiveness of our findings by collaborating across multiple centers to obtain a larger and more diverse sample. Second, notwithstanding our rigorous efforts to ensure the consistency of translation proficiency, the inherent diversity of language expression may still influence the generation by ChatGPT 4.0 and the evaluation by reviewers. Future work may involve refining the translation input instructions to minimize the impact of translation on research outcomes. Third, the quality of responses generated by ChatGPT-4.0 is closely tied to the prompt formulation. Therefore, additional experiments are required to develop more comprehensive and tailored prompts to enhance its clinical utility. Additionally, this study assessed the reports generated by ChatGPT 4.0 from the perspective of healthcare professionals. The evaluation system for ChatGPT 4.0’s ability to produce health examination reports could be further refined by incorporating the readability assessments from the examination clients themselves. Future endeavors may include inviting a cohort of examination clients without medical backgrounds to conduct a more comprehensive evaluation of ChatGPT via questionnaires or similar methods.

With the ongoing learning and iteration of ChatGPT, such as the recent release of the ChatGPT o1 model and ChatGPT5, we are optimistic that its AI capabilities will continue to improve, particularly in the medical domain. Existing studies have demonstrated that the readability of responses to medical questions generated by the latest generation of large language models has been enhanced compared to previous versions. However, further validation in real-world applications is still anticipated to ensure their effectiveness. With the increasing proportion of AI utilization in medical activities, the ability of large language models (LLMs) to address medical or healthcare-related issues is poised to strengthen progressively. This advancement holds the potential to make a more substantial contribution to the field of health management and to facilitate the application and dissemination of such technologies in real-world practice (28).

We believe that the current version of ChatGPT would be more suitable as an assistant in clinical check-up examinations, applicable to both English and Chinese contexts. It is recommended to use ChatGPT to assist in providing clinical guidelines, organizing check-up items, compiling customers’ report results according to different systems, and offering preliminary diagnoses, especially in simpler cases. All responses generated by ChatGPT should be reviewed and finalized by the clinical chief physician, which would significantly enhance the efficiency of clinical physicians and help produce more systematic and accurate check-up reports. And ChatGPT 4.0 is not yet recommended to provide personalized medical suggestions to patients.

This experiment highlights the promising potential of artificial intelligence in check-up examinations. We are confident that ChatGPT will be integrated into clinical practice in the future, potentially taking on significant roles in check-up examination departments.

## Conclusion

In conclusion, ChatGPT 4.0 has demonstrated significant potential in generating check-up reports. Its greatest strength lies in its ability to implement and complete rigorous tasks effectively. It has proven capable of adhering to clinical guidelines, providing accurate diagnoses, systematically summarizing items, and maintaining consistency. However, it has limitations in prioritizing examination items by health risk and offering suitable, individualized medical suggestions. Furthermore, ChatGPT 4.0 did not show a significant advantage in handling clinical cases in either English or Chinese environments. Overall, ChatGPT 4.0 is currently suitable as an assistant to the chief examiner, recommended for completing simpler tasks independently and contributing to specific parts of check-up reports, such as preliminary diagnoses and providing reference medical guidelines. To avoid occasional errors, the content generated by ChatGPT 4.0 should be reviewed by chief examiners before final adoption. With continuous development and progress, the application of ChatGPT in the clinical check-up domain will be further enhanced and optimized. It has the potential to improve medical efficiency, the quality of clinical check-up work, and to provide clients with excellent, patient-centered services.

## Data Availability

The original contributions presented in the study are included in the article/supplementary material, further inquiries can be directed to the corresponding authors.
